# Development of an Open Face Home Cage Running Wheel for Testing Activity-Based Anorexia and Other Applications

**DOI:** 10.1523/ENEURO.0246-22.2022

**Published:** 2022-10-25

**Authors:** Nathan Godfrey, Kehan Chen, Temoor Tayyab, Gina Dimitropoulos, Frank P. MacMaster, Stephanie L. Borgland

**Affiliations:** Hotchkiss Brain Institute, The University of Calgary, Calgary, Alberta T2N 4N1, Canada

**Keywords:** activity-based anorexia, locomotor activity, open behavior, open source, running wheels

## Abstract

Running wheels for mice residing in the home cage are useful for the continuous measurement of locomotor activity for studies testing exercise interventions or exercise-induced effects on brain and metabolism. Here, we have developed an open source, printable, open-faced running wheel that is automated to collect locomotor information such as distance traveled, wheel direction, and velocity that can be binned into epochs over 24 h or multiple days. This system allows for remote data collection to avoid human interference in mouse behavioral experiments. We tested this system in an activity-based anorexia procedure. Using these wheels, we replicate previous findings that food restriction augments wheel-running activity.

## Significance Statement

Anorexia nervosa (AN) is a psychiatric disease with few treatments and a high mortality rate. It is important to better understand the biology to accelerate the development of new therapies. The most used animal model to study AN is the activity-based anorexia model, which measures physical activity during food restriction. We have developed open source running wheels that allow for continuous measurement of activity for multiday experiments and demonstrated efficacy in the activity-based anorexia model.

## Introduction

The prevalence of eating disorders such as anorexia nervosa (AN) has been escalating in recent years (Val-Laillet et al., 2015) and has increased over the covid-19 pandemic (Devoe et al., 2022). AN has a lifetime prevalence of 1% (Hudson et al., 2007; Smink et al., 2012; Bou Khalil et al., 2017). The mortality rate of these individuals is 5 times greater than a healthy individual (Arcelus et al., 2011; Bou Khalil et al., 2017) which is the highest mortality rate for any mental disorder (Arcelus et al., 2011; Smink et al., 2012; Val-Laillet et al., 2015). Furthermore, the current recovery rate for AN, 10 years after onset, is a meager 10% (Bergh et al., 2013). An improved understanding of the etiology and neurobiological underpinnings of this disorder will lead to improved treatments and outcomes.

The hallmark characteristics of AN are a restriction in energy intake that leads to low body weight, an intense fear of gaining weight, and a disturbed body image including overestimating body size (American Psychiatric Association, 2013). In addition, an increase in physical activity has been observed among many individuals with AN (Bergh et al., 2013). A common model used to study AN in rodents is the activity-based anorexia (ABA) model that replicates many symptoms seen in AN (Routtenberg, 1968; Watanabe et al., 1992; Bergh et al., 2013; Gutierrez, 2013). In this model, access to a running wheel is paired with food restriction (FR), resulting in an increase in activity, a decrease in food consumption, and a reduction in body weight. Mice and rats lose the ability to self-regulate their food intake and energy expenditure, eventually resulting in weight loss to the humane end point where they are removed from the study.

An important limitation to the ABA model is easy access to mouse running wheels with the ability to record mouse activity. Although commercial products are available, they are expensive and require proprietary software devoted to wheel use (Welch et al., 2018). Many commercial products also use a closed wheel that is not compatible with modern optical and recording techniques. Other open source wheel designs had features that were not ideal for our application (Bivona and Poynter, 2021; Zhu et al., 2021). For these reasons, we designed and constructed an open source running wheel system that runs independent from a central computer. The wheels are 3D printed and are operated using a Raspberry Pi zero W, a small but highly available microprocessor, and are programmed in Python. The data are transmitted to a personal computer via e-mail, where it is then automatically downloaded, parsed, and analyzed with Python and MATLAB programs. Our running wheel system is inexpensive, simple, adaptable, and completely open source. In this study, we demonstrated its utility with the ABA model.

## Materials and Methods

### Mice

Thirty-two female BALB/c mice (Charles River) 7–8 weeks old were used for the ABA model. Mice acclimatized in the animal facility 3–5 d before the habituation period of the study. Before habituation, mice were housed in groups of 2–5, maintained on a 12 h light/dark schedule [lights on at 0800 h, zeitgeber time 0 (ZT0)], and given chow and water *ad libitum* (*Ad lib*). Mice were fed standard chow (Pico-Vac Mouse Diet 5062, Lab Supply), which is composed of the following (percentage of total kcal): 23% protein, 22% fat (ether extract), and 55% carbohydrate. The total density of this diet was 4.60 kcal/g. All experiments and procedures were performed in accordance with the ethical guidelines established by the Canadian Council for Animal Care and were approved by the University of Calgary Animal Care Committee (protocol no. AC21-0034).

### Running wheels

**Figure 1.**
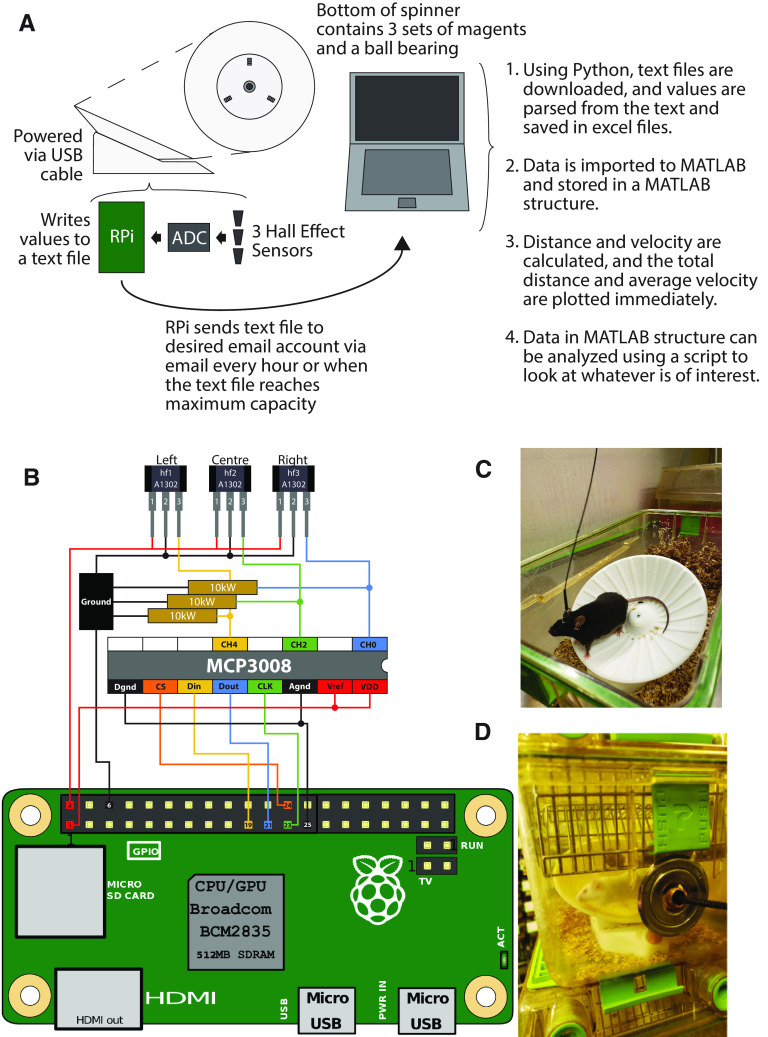
Description of the running wheels and computer interface. ***A***, Illustration indicating how data are collected and distance and velocity are calculated. ***B***, Illustration of the Raspberry Pi motherboard connections. Image of the Raspberry Pi computer is adapted from https://en.wikipedia.org/wiki/Raspberry_Pi. ***C***, Photograph of a mouse on the running wheel when connected to a fiber-optic patch cord. ***D***, Photograph of a Green Line cage containing a mouse on the wheel with the power cord attached through the bottle holder. Water bottles are delivered via the food hopper for these experiments. Additional photographs of the wheel are located at https://github.com/borglandlab/RunningWheel.

Each running wheel consists of a 3D printed wheel and electrical components connected to a Raspberry Pi 0 W (Fig. 1*A*; www.PiShop.ca). The spinner part of the wheel contains a metal ball bearing at the center and is surrounded by three sets of magnets that are evenly spaced. Three hall effect sensors that protrude up through the base of the wheel detect changes in the magnetic field, which occurs when the magnets pass over top. Each hall effect sensor has three pins, with the first pin being connected to the 3.3 V Raspberry Pi power supply through the MCP3008 analog digital converter (ADC; Fig. 1*A*,*B*). The second pin connects to the Raspberry Pi ground. The third pin transmits magnetic field values to the Raspberry Pi through the ADC. To reduce noise, a 10 kΩ pull-up resistor is placed between the third pin and the ground. The ADC is soldered to a piece of printed circuit board (PCB) prototyping board and 20 gauge solid wires connect the board to the Raspberry Pi and the three hall effect sensors. The three hall effect sensors are inserted inside a 3D printed sensor holder, which fits through a gap left in the base of the wheel. All Raspberry Pi boards receive power through the micro-USB port. Copper tubing was bent and cut to serve as a protective sleave for the USB power cable inside of the mouse cage. The total cost of each wheel was approximately $65.00 CAD (Canadian dollars).

### 3D printing

All files for spinner parts were made as SLDPRT (SolidWorks Part) files in solid works and then converted to STL (stereolithography) files. Both SLDPRT and STL files are available at (https://github.com/borglandlab/RunningWheel) to allow for direct printing or modifying the wheel to individual needs. When assembled, the wheel is 15.24 cm (6 inches) I width, 10.64 cm (4.19 inches) in height, and 14.45 cm (5.69 inches) in depth. Using the STL files, 3D models were then sliced using Cura 4.9 in preparation for printing (https://ultimaker.com/learn/ultimaker-cura-4-9-seamless-and-efficient-with-digital-library-integration). For the wheels used in the ABA model, printing was done using either a Stock Eryone Thinker ER20 (ShenZhen Eryone Technology Co, Ltd) or FLSUN QQ-S-pro (Zhengzhou Chaokuo Electronic Co, Ltd) with 0.4 mm E3D V6 nozzle. All 3D printing used generic 1.75-mm-diameter PLA (polylactic acid). To demonstrate the universal nature of our design, all wheel parts were also sliced and printed with the Sindoh 3DWOX printer (Sindoh Co, Ltd) and supporting software with excellent results. More information on the printing process can be found within our 3D model files.

### Raspberry Pi code

The Raspberry Pi is programmed to continuously check the values of all three hall effect sensors. When the value of one hall effect sensor is >30, the trigger time and values for all three hall effect sensors are recorded. New values cannot be recorded until the values for all three hall effect sensors drop to <15, allowing the magnet to clear to ensure that the next trigger is indeed a separate incident. Thus, a single magnet passing over the three sensors will not accidently trigger the sensors more than once. For this reason, because there are magnets in three different locations on the spinner top, each triggering of the hall effect sensors reliably indicates that the mouse has traveled one-third of the circumference at the running position of the spinner top. Every hour, or when the number of recorded entries is >3000, the entries are written to a text file. These data will be stored on the micro-secure digital (SD) card and can be accessed at the end of the experiment. Although writing to the text file is relatively fast, the Raspberry Pi will wait for a moment when no wheel movement is occurring to ensure that minimal data are lost during this process. In addition, if the Raspberry Pi detects an Internet connection with speeds >1 Mb/s it will also send an e-mail containing the text file information to the specified e-mail account. The frequency of data storage and transmittal can be adjusted for specific experimental needs (Fig. 1*A*). To connect the Raspberry Pi boards to the university network, we supplied the MAC (media access control) addresses from each Raspberry Pi to the IT services.

### Personal computer

Using Python code (https://github.com/borglandlab/RunningWheel), the e-mails sent from the Raspberry Pi are downloaded (Fig. 1*A*,*B*). Trigger times and values for each hall effect sensor are parsed from the text, organized, and stored in a MS Excel workbook. Individual emails are stored as sheets within a workbook, with a maximum of 49 sheets per workbook before a new workbook is created. Each running wheel is assigned a folder that will contain all the excel workbooks for that specific wheel. A directory MS Excel workbook is also created to keep track of the location of each sheet. A MATLAB program imports the data from these excel workbooks and stores each individual time point, direction of wheel rotation, the cumulative distance, and the velocity for all running wheels in a MATLAB structure (www.Mathworks.com). MATLAB will also immediately create graphs showing the total distance and average velocity of the wheels. This code is run from a Python GUI (graphical user interface; www.Python.org), automatically taking the data from a text file in your e-mail account to an organized MATLAB structure and viewable graphs. The MATLAB structure can be further analyzed by the code provided or by your own personal analysis. Furthermore, data can be binned into daily and hourly time frames, making analysis and data visualization more versatile (Fig. 1).

In addition to data collected from the running wheel, we also recorded body weight, food, and water weight each day on a workbook stored in Dropbox (www.Dropbox.com). This allowed us to automatically calculate changes in mouse weight, changes in food and water consumption, and the removal from study threshold with MATLAB. Graphs were automatically generated. Like the code for the running wheel, this can also be run from our Python GUI. However, because this code is specific to our experiment, it has been made optional to run in the experiment.

### Activity-based anorexia model

**Figure 2.**
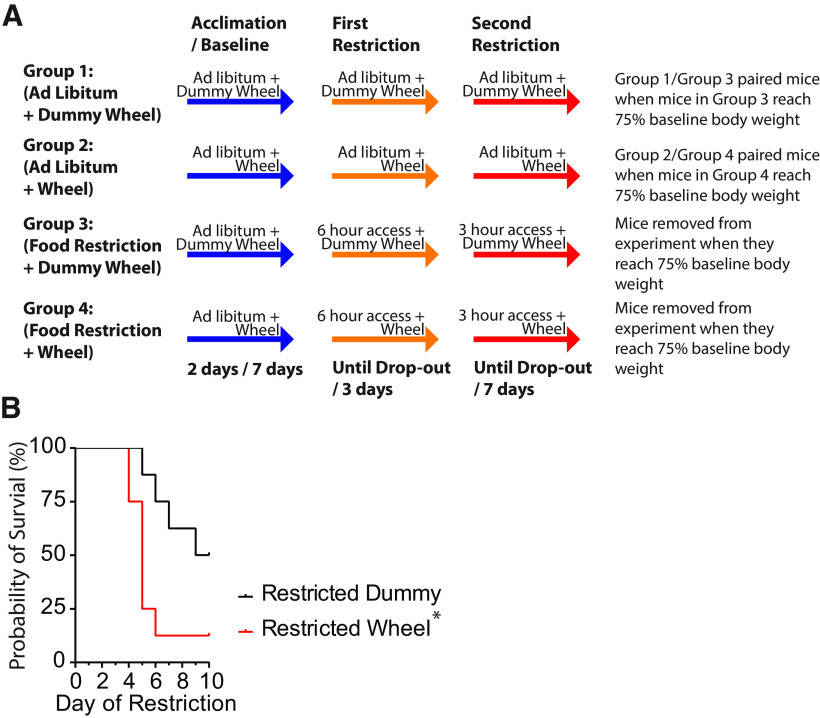
Mice on an activity-based anorexia model with access to a running wheel have decreased probability of survival when food restricted. ***A***, Description and time course of the following four groups: (1) *Ad lib* + dummy, (2) *Ad lib* + wheel, (3) FR + dummy, and (4) FR + wheel. ***B***, FR mice with access to a running wheel (red) are removed from the study sooner when food restricted compared with *Ad lib* mice with a running wheel (black). Extended Data Figure 2-1 indicates that mice did not lose weight to the humane end point when on 6 h food restriction for up to 10 d. As such, we added a 3 h food restriction after 3 d of 6 h food restriction.

The ABA model used in this study was based on models previously published (Ho et al., 2016; Achamrah et al., 2017; Welch et al., 2018). Mice were placed in individual cages that contained a spinning or nonspinning (dummy) running wheel, cardboard shelter, water bottle, and *ad libitum* chow in a feeding hopper, and were left uninterrupted for 48 h to habituate to the novel cage. A 7 d baseline period immediately followed the habituation period. During the baseline, body weight, food, and water were weighed each day at ZT 0130 h. The spinner tops were cleaned, and 8–10 g of new food was weighed and left in the food hopper. Following baseline, mice were split into the following four groups: (1) *ad libitum* access with dummy wheel (*n* = 8); (2) *ad libitum* access with running wheel (*n* = 8); (3) food restriction with dummy wheel (*n* = 8); and (4) food restriction with running wheel (*n* = 8). Group 1 was paired with group 3, and group 2 was paired with group 4, such that measurements could be compared with a time-matched control. During this period, groups 3 and 4 were given access to food for 6 h (ZT 0130 h to ZT 0730 h) for 3 d (days 8–10). In a pilot study, mice did not lose weight to the humane end point when on 6 h food restriction for up to 10 d (Fig. 2*B*, Extended Data Fig. 2-1); therefore, we modified the protocol so that after 3 d of 6 h food restriction, they were food restricted to 3 h (ZT 0130 h to ZT 0430 h) for the next 6 d (days 11–17; Fig. 2*A*). Body weight, food, and water were weighed daily at ZT 0130 h. Spinner tops were cleaned at ZT 0130 h for nonrestricted mice, and after food restriction for restricted mice. Restricted mice, and their paired controls, were removed from the study if their body weight fell to 75% of their body weight recorded on the last day of baseline. Daily measurements of distance and velocity were recorded from ZT 1700 h (12:00 A.M.) to ZT 1700 h the next day. The anticipatory activity was measured in the 3 h before food access. Activity during food intake was measured during first 3 h of food access. Activity during the postprandial period was measured ZT 0730 h to ZT 1030 h. Mice begin to be removed from the experiment during day 11, and by day 13, after 6 d of food restriction, only one mouse remained in groups 2 and 4, obscuring comparisons beyond this point.

10.1523/ENEURO.0246-22.2022.f2-1 Figure 2-1 FR Mice with access to a running wheel lose body weight but adapt food intake. ***A***, Time course of daily body weight measurements taken at 9:00 A.M. each day during baseline and the 6 h food restriction. ***B***, Body weight measurements from *Ad lib* (open bars, *n* = 4) or FR (filled bars, *n* = 4) mice with access to a dummy wheel or running wheel on day 11, the time point before one mouse was removed from the study due to dermatitis. Body weight was less in restricted mice (*t*_(6)_ = 4.13, *p* = 0.0061). ***C***, Time course of food consumption each day during baseline, after the 6 h FR period. ***D***, Food intake over the 24 h period on day 11 from *Ad lib* (open bars, *n* = 4) and FR (filled bars, *n* = 4) mice was decreased in restricted mice (*t*_(6)_ = 4.65, *p* = 0.0035). ***E***, Daily distance traveled on the running wheel in *Ad lib* (open circles, *n* = 4) or FR (filled circles, *n* = 4) mice. ***F***, Averaged distance traveled over 24 h measured on day 11 from *Ad lib* (open bars) or FR (filled bars) mice was not different between groups (*t*_(6)_ = 2.4, *p* = 0.053). Bars represent the mean ± SEM. Symbols represent individual mice. Download Figure 2-1, EPS file.

### Data analysis

All values are expressed as the mean ± SEM and assessed for normality using a Shapiro–Wilk test. Statistical significance was assessed by using two-tailed unpaired Student’s *t* test for two comparisons. A two-way ANOVA followed by Sidak multiple-comparisons test was used for multiple group comparisons. GraphPad Prism 8.3 (GraphPad Software) was used to perform statistical analysis. Significance was defined at alpha = 0.05, *p* < 0.05 *, *p* < 0.01 **, *p* < 0.001 ***, *P* < 0.0001 ****.

## Results

To validate the utility of our 3D-printed running wheels, we conducted a 3-week-long ABA model. During the 7 d baseline period, mice had access to active or inactive (dummy) running wheels and *ad libitum* access to food and water. On the final day of baseline, body weight was compared across the four groups: *Ad lib* access with dummy wheel (17.7 ± 0.2 g); *Ad lib* access with running wheel (17.4 ± 0.3 g); FR with dummy wheel (17.7 ± 0.3 g); and FR with running wheel (18.0 ± 0.3 g). There was no main effect of wheel running or food on body weight (running effect: *F*_(1,28)_ = 0.003, *p* = 0.9; food effect: *F*_(1,28)_ = 1.0, *p* = 0.3), or wheel running × food interaction (interaction: *F*_(1,28)_ = 0.8, *p* = 0.4).

To examine the effect of FR on body weight, food and water consumption, and wheel running, we measured these parameters on day 11, the first day of the second food restriction phase. This was the time point when some mice first reached the humane end point and were removed from the study. The number of days to reach the humane end point whereby mouse body weight dropped to 75% of body weight from that measured on the last day of baseline was compared between FR with a running wheel and FR with a dummy wheel. FR mice exposed to wheel running had reduced probability of survival compared with FR mice with the dummy wheel (log-rank test: χ^2^ = 5.1; df = 1; *p* = 0.02; Fig. 2*B*). Thus, FR mice with access to a running wheel are removed from the experiment earlier.

**Figure 3.**
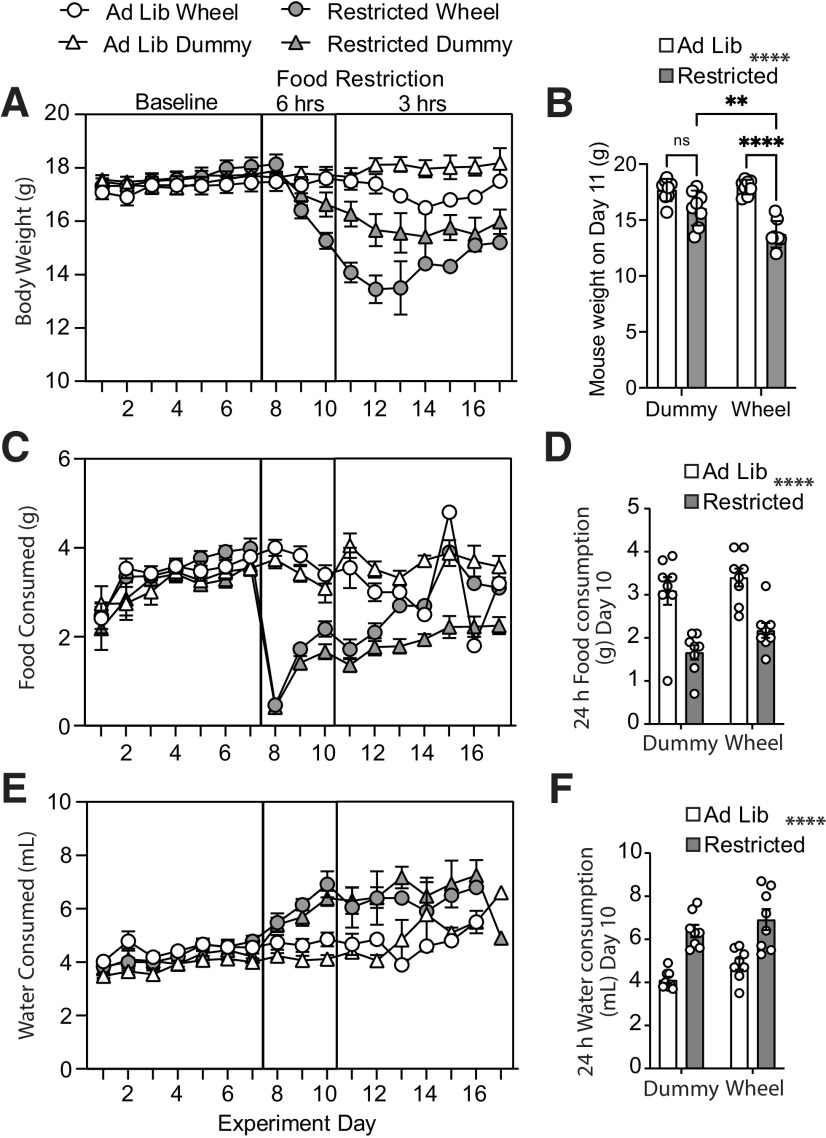
FR Mice with access to a running wheel lose more body weight. ***A***, Time course of daily body weight measurements taken at 9:00 A.M. each day during baseline, 6 h food restriction, and then 3 h food restriction. ***B***, Body weight measurements from *Ad lib* (open bars) or FR (filled bars) mice with access to a dummy wheel or running wheel on day 11, the time point before some mice were removed from the study. ***C***, Time course of food consumption each day during baseline, after the 3 h FR period or after the 6 h FR period. ***D***, Food intake over the 24 h period preceding the time point before some mice were removed from the study from *Ad lib* (open bars) and FR (filled bars) mice. ***E***, Daily water consumption during baseline, 3 h FR, and 6 h FR. FR increased water consumed in both groups regardless of access to the running wheel. ***F***, Water consumption over the 24 h period preceding the time point before some mice were removed from the study from *Ad lib* (open bars) and FR (filled bars) mice that have access to a dummy or running wheel. Bars represent the mean ± SEM. Symbols represent individual mice.

To determine the effect of FR, mouse body weight was compared between groups on day 11. FR running wheel mice were at 78.1 ± 1.5% of their baseline body weight, whereas *Ad lib* dummy wheel mice were at 99.7 ± 0.9%, *Ad lib* running wheel mice were at 100 ± 0.8%, and FR dummy wheel mice were at 91.7 ± 1.9% of their original body weight. There were main effects of running and FR (running effect: *F*_(1,28)_ = 9.8, *p* < 0.004; food restriction effect: *F*_(1,28)_ = 40.9, *p* < 0.0001) as well as a running × FR interaction (interaction: *F*_(1,28)_ = 7.3, *p* = 0.01). A Sidak multiple-comparisons test showed that FR running wheel mice lost more weight than FR dummy wheel mice (*p* = 0.002) and *Ad lib* running wheel mice (*p* < 0.0001; Fig. 3*A*,*B*).

Daily food consumption was compared between groups (Fig. 3*C*,*D*). Twenty-four hour food consumption from day 10 demonstrated a main effect of FR (food restriction effect: *F*_(1,28)_ = 34.6, *p* < 0.0001) but no effect of running (running effect: *F*_(1,28)_ = 3.4, *p* = 0.07) on food consumption. Given that both wheel and dummy groups had reduced food consumption during the FR period, there was no interaction (*F*_(1,28)_ = 0.2, *p* = 0.7). However, a Sidak *post hoc* test on the main effect of food restriction indicated significant reductions in food intake in both dummy wheel groups (*Ad lib* dummy wheel mice, 3.1 ± 0.3 g; vs FR dummy wheel mice, 1.7 ± 0.2 g; *p* = 0.0002) and running wheel groups (*Ad lib* running wheel mice: 3.4 ± 0.2 g; vs FR running wheel mice, 2.2 ± 0.2 g; *p* = 0.001; Fig. 3*D*). Thus, access to the running wheel did not further restrict food intake in the FR groups.

We next measured daily water consumption (Fig. 3*E*,*F*). On day 10, there was a main effect of FR on 24 h water consumption (food restriction effect: *F*_(1,28)_ = 46.4, *p* < 0.0001) but no effect of running on water consumption (running effect: *F*_(1,28)_ = 3.9, *p* = 0.06) or running × FR interaction (interaction: *F*_(1,28)_ = 0.1, *p* = 0.7). A Sidak *post hoc* test on the main effect of food restriction indicated a significant increase in water intake in both dummy wheel groups (*Ad lib* dummy wheel mice: 4.1 ± 0.2 ml; vs FR dummy wheel mice: 6.4 ± 0.3 ml, *p* < 0.0001) and running wheel groups (*Ad lib* running wheel mice: 4.8 ± 0.3 ml; vs FR running wheel mice: 6.9 ± 0.5 ml; *p* = 0.0002; Fig. 3*F*). Together, these data indicate that FR mice with access to a running wheel have reduced body weight compared with FR mice without the running wheel. However, food and water intake between these groups was similar.

**Figure 4.**
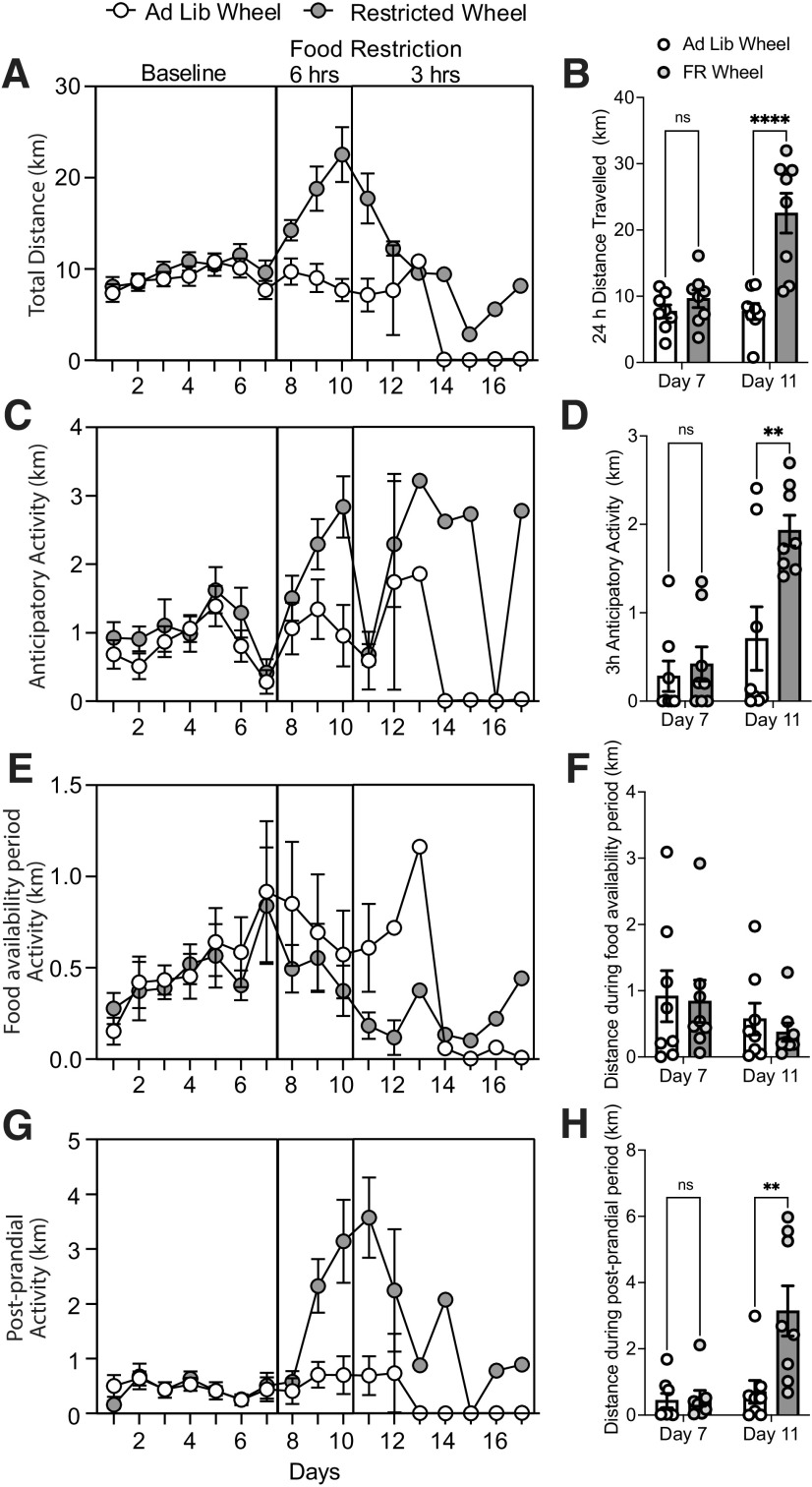
Distance traveled increases with food restriction. ***A***, Daily distance traveled on the running wheel in *Ad lib* (open circles) or FR (filled circles) mice. ***B***, Averaged distance traveled over 24 h measured on day 7 or day 11 from *Ad lib* (open bars) or FR (filled bars) mice. ***C***, Daily anticipatory activity measured within the 3 h period before food delivery in *Ad lib* or FR mice. ***D***, Averaged 3 h anticipatory activity measured on day 7 or day 11 from *Ad lib* (open bars) or FR (filled bars) mice. ***E***, Daily distance traveled measured during the 3 h food availability period from *Ad lib* or FR mice. ***F***, Averaged distance traveled during the first 3 h of the food availability period on day 7 or day 11 from *Ad lib* or FR mice. ***G***, Time course of daily postprandial activity over 3 h after food access from *Ad lib* or FR mice. ***H***, Averaged 3 h postprandial distance traveled on day 7 or day 11 from *Ad lib* or FR mice. Bars represent the mean ± SEM. Symbols represent individual mice.

We next recorded daily 24 h locomotor activity of *Ad lib* and FR mice with access to our 3D printed running wheels and supporting Python/MATLAB code (Fig. 4). Distance traveled between *Ad lib* and FR mice was measured daily. There were eight mice per group until day 11 when mice were removed from the study because of low body weight, and thus the number of animals per group varied on subsequent days (day 11, *n* = 6; day 12–16, *n* = 1; Fig. 4*A*). Thus, the averaged distance traveled during food restriction was measured the morning of day 11, which accounted for the preceding 24 h period before mice were removed from the study. There was a main effect of food restriction (*F*_(1,28)_ = 21.1, *p* < 0.0001) and a main effect of time (*F*_(1,28)_ = 12.5, *p* = 0.001), and a time × food restriction interaction (*F*_(1,28)_ = 12.5, *p* = 0.001). A Sidak *post hoc* test indicated no difference during the baseline period (day 7), but a significant increase in distance traveled after food restriction (*Ad lib* groups, 7.7 ± 1.2 km; vs FR groups, 22.5 ± 3.0 km; *p* < 0.0001; Fig. 4*B*). Thus, FR increases wheel running in mice.

We next examined whether FR influences daily anticipatory activity in the 3 h period before food delivery (Fig. 4*C*,*D*). There was a main effect of food restriction (*F*_(1,28)_ = 8.2, *p* = 0.008), a main effect of time (*F*_(1,28)_ = 16.5, *p* = 0.0003), and a significant food restriction × time interaction (*F*_(1,28)_ = 5.2, *p* = 0.03). A Sidak *post hoc* test indicated no significant difference during baseline (day 7; *p* = 0.9), but it did indicate a significant increase in anticipatory activity during food restriction (day 11; *Ad lib* groups, 0.9 ± 0.4 km; vs FR groups, 2.8 ± 0.4 km; *p* = 0.002; Fig. 4*D*). Thus, FR increases anticipatory wheel running.

Activity during the first 3 h of the food availability period was not different between groups (*Ad lib* wheel mice, 0.6 ± 0.2 km; FR wheel mice: 0.4 ± 0.1 km). There was no main effect of food restriction (*F*_(1,28)_ = 0.2, *p* = 0.6), time (*F*_(1,28)_ = 2.0, *p* = 0.2), or food restriction × time interaction (*F*_(1,28)_ = 0.04, *p* = 0.8; Fig. 4*E*,*F*). These results suggest that, in our procedure, FR mice are making choices for food over wheel running during food availability.

We next examined daily wheel running activity during the postprandial period (Fig. 4*G*). There was a main effect of food restriction (*F*_(1,28)_ = 7.9, *p* = 0.009), a main effect of time (*F*_(1,28)_ = 10.5, *p* = 0.003), and a significant food restriction × time interaction (*F*_(1,28)_ = 7.1, *p* = 0.01). A Sidak *post hoc* test indicated that while postprandial activity was not different between groups during the baseline (*p* = 0.99), in the 3 h period after food access, the postprandial activity was greater in FR mice (3.1 ± 0.7 km) than in *Ad lib* mice (0.7 ± 0.3 km, *p* = 0.001; Fig. 4*H*). Thus, FR mice increase their activity in the period after food availability compared with *Ad lib* mice.

**Figure 5.**
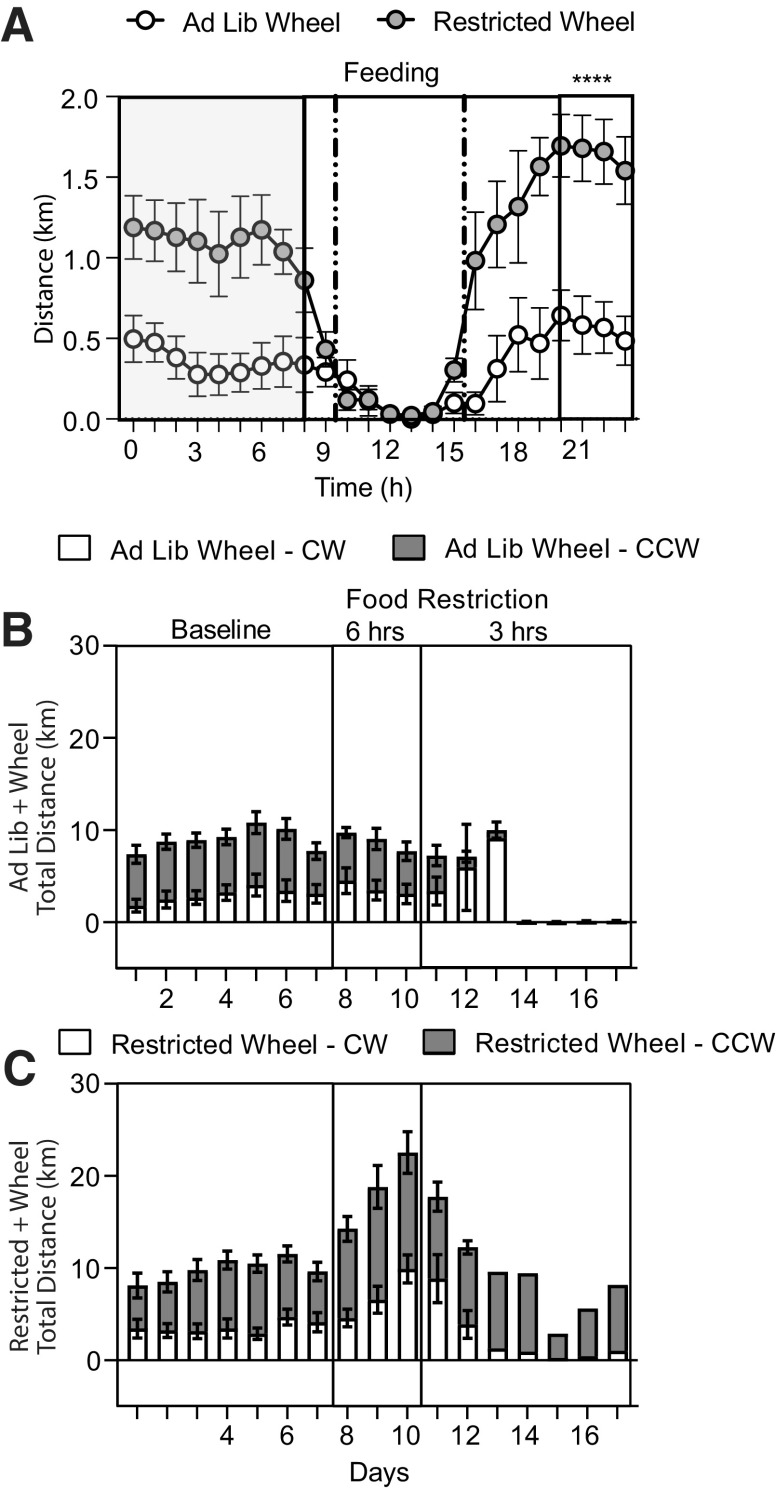
Circadian pattern of wheel running. ***A***, Over a 24 h period, hourly wheel running is enhanced in FR mice (filled circles) compared with *Ad lib* mice (open circles) except during the food availability period. This data were recorded on day 10. Shaded boxes represent the dark cycle. Hashed lines represent the food availability period. ***B***, Daily wheel running in *Ad lib* mice on day 10 as identified by clockwise running (open bars) and counterclockwise running (filled bars). ***C***, Daily wheel running in FR mice on day 10 as identified by clockwise running (open bars) and counterclockwise running (filled bars). Bars represent the mean ± SEM. Symbols represent individual mice.

To examine the circadian pattern of activity, we compared the hourly distance traveled of *Ad lib* or FR mice binned by hour and plotted across all of day 10 from 12:00 A.M. to day 11 at 12:00 A.M. There was a main effect of food restriction (*F*_(1,14)_ = 20.9, *p* = 0.0004), a main effect of time (*F*_(4.06,58.84)_ = 13.05, *p* < 0.0001), and a time × food restriction interaction (*F*_(23,322)_ = 4.03, *p* <0.0001). A Sidak *post hoc* test revealed significant differences in activity between *Ad lib* and FR groups in hours 18–24, the early part of their dark cycle (Fig. 5*A*). Furthermore, to demonstrate the full utility of the running wheels, the total daily activity of both *Ad lib* and food-restricted mice was plotted, showing the direction of the rotation of the wheels [i.e., clockwise (CW) or counterclockwise (CCW); Fig. 5*B*,*C*]. We recorded wheel rotations in CW or CCW from *Ad lib* and FR mice, with a greater amount of running in the CCW wheel direction. Together, FR mice travel greater distances on the running wheels over a 24 h period.

**Figure 6.**
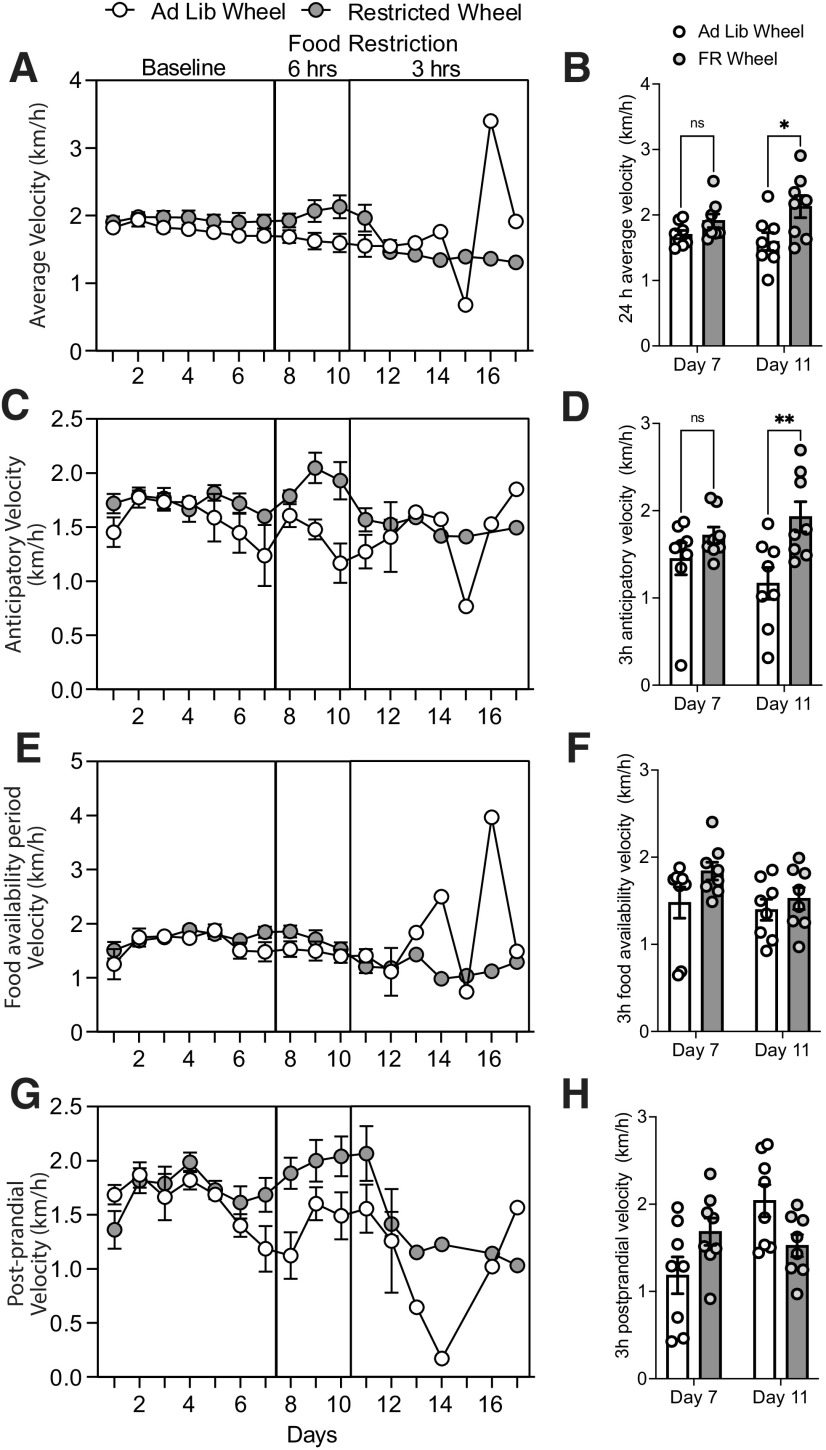
Averaged velocity is greater in FR mice. ***A***, Daily velocity on the running wheel in *Ad lib* (open circles) or FR (filled circles) mice. ***B***, Averaged velocity over 24 h measured on day 7 or day 11 from *Ad lib* (open bars) or FR (filled bars) mice. ***C***, Daily velocity measured within the 3 h period before food delivery in *Ad lib* or FR mice. ***D***, Averaged 3 h anticipatory velocity measured on day 7 or day 11 from *Ad lib* (open bars) or FR (filled bars) mice. ***E***, Daily velocity measured during the 3 h food availability period from *Ad lib* or FR mice. ***F***, Averaged velocity during the first 3 h of the food availability period on day 7 or day 11 from *Ad lib* or FR mice. ***G***, Time course of daily postprandial velocity over 3 h after food access from *Ad lib* or FR mice. ***H***, Averaged 3 h postprandial velocity on day 7 or day 11 from *Ad lib* or FR mice. Bars represent the mean ± SEM. Symbols represent individual mice.

Given the high sensitivity of these running wheels, we calculated the average daily velocity of the mice from the recorded data (Fig. 6*A*). When comparing the baseline (day 7) to the final day before some mice were removed from the study (day 11), we found a main effect of food restriction (*F*_(1,28)_ = 8.9, *p* = 0.006), but no main effect of time (*F*_(1,28)_ = 0.2, *p* = 0.7) or significant time × food restriction interaction (*F*_(1,28)_ = 1.7, *p* = 0.2). We performed Sidak *post hoc* tests on the main effect of food restriction and found no significant difference on the baseline day 7 (*Ad lib* groups, 1.7 ± 0.1 km/h; vs FR groups, 1.8 ± 0.1 km/h; *p* = 0.4), but a significant increase in velocity after food restriction (*Ad lib* groups, 1.5 ± 0.1 km/h; vs FR groups, 2.1 ± 0.2 km/h; *p* = 0.01; Fig. 6*B*). Similarly, for the 3 h anticipatory velocity, there was a main effect of food restriction (*F*_(1,28)_ = 10.1, *p* = 0.004), but no main effect of time (*F*_(1,28)_ = 0.04, *p* = 0.8) or time × food restriction interaction (*F*_(1,28)_ = 2.3, P 0.1). A Sidak *post hoc* test on the main effect of food restriction revealed a significant difference after FR (*Ad lib* groups, 1.1 ± 0.2 km/h; vs FR groups, 1.9 ± 0.2 km/h; *p* = 0.005), but not during baseline (*Ad lib* groups, 1.4 ± 0.2 km/h; vs FR groups, 1.7 ± 0.1 km/h; *p* = 0.4; Fig. 6*C*,*D*). During the food availability period, there were no main effects of food restriction (*F*_(1,28)_ = 3.3, *p* = 0.08), time (*F*_(1,28)_ = 2.2, *p* = 0.2), or food restriction × time interaction (*F*_(1,28)_ = 0.7, *p* = 0.4; *Ad lib* groups, 1.4 ± 0.1 km/h; vs FR groups, 1.5 ± 0.1 km/h; Fig. 6*E*,*F*). Finally, during the 3 h postprandial period, there were no main effects of food restriction (*F*_(1,28)_ = 0.001, *p* = 0.9) or time (*F*_(1,28)_ = 4.1, *p* = 0.05) on velocity (*Ad lib* groups, 2.0 ± 0.2 km/h; vs FR groups, 1.5 ± 0.1 km/h; Fig. 6*G*,*H*). Together, this running wheel platform can also provide measurements of velocity, and we demonstrate that FR mice also have increased velocity during the anticipatory period.

## Discussion

Our team has designed and built an open source running wheel system and validated the utility of our system through the ABA model. There are several advantages to our running wheels. First, they are highly economical compared with commercial products. The total cost to produce our wheels is approximately $65 CAD, and that production uses components that are readily available. This also means that replacement parts can be made should mice damage a part during the experiment. Second, the design of our running wheels makes them suitable for a variety of experiments and cage types. Like commercial and other open source products, these wheels have a low-profile design, making them ideal for mouse cages with low lids. Also, because of the open top design of the spinner these wheels are compatible with both optogenetics and fiber photometry. Third, with three sensors and three magnet locations, our running wheels can be used to reliably monitor the distance run by mice and monitor details such as wheel direction and speed. This increases their utility as a tool for data collection. Fourth, this system is adaptable for any laboratory without the need for an expensive computer and software for operation and data acquisition. These wheels only need power and an Internet connection. Even a good Internet connection has moments of instability, therefore these running wheels are built with safeguards to test the Internet connection before downloading data. The activity of the mice can then be monitored using our Python and MATLAB programs on your personal computer so that the experimenter is not in the room influencing the activity of the mice. Fifth, these running wheels can be replicated even without advanced expertise in electronics and computer science. All wheel parts are print ready. However, we have included SLDPRT files, which can be modified to your specific application. Although soldering is required for connecting the electrical components, the use of the PCB prototyping board makes this process straightforward. In addition, all the code to run this system, from the Raspberry Pi to your personal computer, is written in either Python or MATLAB, making this system adaptable to fit your specific needs. Furthermore, versions of this code are provided for both the macOS and Windows operating system.

Our running wheels have three limitations that are the result of design choices made to improve utility. First, our running wheels are not battery powered like some commercial products, but instead require connection to a USB power cable. This choice was made to avoid the need for battery changes throughout a long-term experiment, which we felt would become more disruptive. Our ABA protocol lasts for ∼3 weeks. Avoiding the problem of dead batteries also avoids the potential of lost data. In addition, using an external power supply also allowed us to design a system that favors data collection speed and precision over energy efficiency. The USB power cable allows for utility despite the battery limitation.

Second, an added feature of our running wheels is their ability to autonomously collect and transfer data via e-mail in addition to saving the data to an SD card. This design removes the need for a nearby computer to act as a hub for data collection. However, according to our tests, this design requires a reliable Internet connection and Internet speeds of ≥1 mb/s for transmitting data via e-mail. However, if the Internet connection drops to <1 mb/s, we have programmed a fail-safe, such that our running wheels will determine whether the Internet speed is >1 mb/s before attempting to transmit an e-mail. If the Internet speed is too slow, the data will be saved only to the SD card and no e-mail will be sent. This is essential since attempting to transmit an e-mail when the Internet speed is too slow can result in the system freezing and data collection being interrupted. In most circumstances, this safeguard will prevent the Raspberry Pi from freezing. For the rare occurrence that the Raspberry Pi either freezes or becomes disconnected from the power source, our data download code also checks to see whether an e-mail has been sent from each running wheel in the past 3 h, sending an alert e-mail to your personal e-mail if this has not occurred. However, even with these safeguards in place, if the Internet connection is unreliable or <1 mb/s, we recommend using the no-Wi-Fi version of the spinner code that we have provided.

Third, our running wheels do not transfer data in real time. This means that, without modifications, they are not designed to be used if your need is to visualize mouse activity each second as it happens. In our design, the running wheel instead sends data either after 1 h or when the stored file reaches a specified capacity of 3000 entries. We found that this approach was more reliable in long-term multiday experiments. Together, our design is intended for long-term data collection of mouse activity with hourly data visualization.

Similar to other low-cost open source running wheel options (Mayr et al., 2020; Bivona and Poynter, 2021; Edwards et al., 2021; Zhu et al., 2021), our system offers a small, low-profile wheel compatible with almost any murine cage (for our facility: Green Line cages, Tecniplast) and data storage on micro-SD cards that tracks distance, running time, and velocity. Using a magnet and hall effect sensors appears to be a common mechanism to record wheel movement (Mayr et al., 2020; Bivona and Poynter, 2021; Edwards et al., 2021), although some designs have used a magnet detected by a reed switch (Zhu et al., 2021). With three hall effect sensors, we and others (Mayr et al., 2020; Bivona and Poynter, 2021) are able to record wheel directionality. Other wheels have features that we did not require for our experiments, such as wheel locking to limit running activity (Mayr et al., 2020; Edwards et al., 2021) and an RFID (radio-frequency identification) reader (Mayr et al., 2020), as our experiments required unlimited running and individual housing with food restriction. Further, we used a wired power setup as we were concerned about battery failure over the long duration of our experiments. Although other systems claimed that the lithium batteries could last up to a month, this was not tested directly (Zhu et al., 2021). One critical difference with our system compared with others is that we used a low-cost Raspberry Pi single-board computer instead of microcontroller feathers (mainboards) such as those made by Adafruit (Zhu et al., 2021) or Arduinos (Mayr et al., 2020; Bivona and Poynter, 2021; Edwards et al., 2021). While those systems have reduced power draw, the Raspberry Pi offers increased flexibility in customizability and programmability using common programming languages such as Python. Together, there are a variety of open source running wheels available that have their advantages and disadvantages. Our system offers another option with increased flexibility in programming.

To verify that this application works using the ABA model, we have demonstrated that, consistent with other studies, FR mice have increased distance traveled, anticipatory, and postprandial locomotor activity compared with the nonrestricted control mice (Klenotich and Dulawa, 2012; Chowdhury et al., 2015; Beeler and Burghardt, 2021). While this did not alter self-induced food restriction, it did lead to a greater weight loss that restricted controls, suggesting that they could no longer match their food intake with their energy needs. Our model used a progressive food restriction of food availability. This method allowed for prolonging the activity of mice in our experiment and to delay the severe reductions in activity because of a loss of energy requirements. Given the reductions in quality of life, high mortality rate, and lack of effective treatments for AN, new models, such as ours are needed to explore the neurobiological underpinnings of this disease and to identify novel therapeutic targets.

In conclusion, we have developed a running wheel and running wheel system that is open source, economical, and highly versatile. Access to these running wheels will increase the ability of other laboratories to do research on AN, leading to improved treatments and outcomes. In addition, there are numerous additional experiments where these wheels can be implemented. Running wheel activity can be used to assess stress, hyperactivity, exercise-induced plasticity, and disruptions in circadian cycles. All of these are important aspects of numerous mental illnesses, increasing the translatability of experimental results.

## Synthesis

Reviewing Editor: Alexxai Kravitz, Washington University in St. Louis Decisions are customarily a result of the Reviewing Editor and the peer reviewers coming together and discussing their recommendations until a consensus is reached. When revisions are invited, a fact-based synthesis statement explaining their decision and outlining what is needed to prepare a revision will be listed below. The following reviewer(s) agreed to reveal their identity: NONE.

Essential comments to be addressed in a revision:

- The significance statement should be improved to highlight the specific problem being solved by this wheel.

- The manuscript needs an IACUC (or similar) statement regarding ethical animal use.

- Photos of the device should be added to Figure 2, ideally within a cage so a reader can easily understand its dimensions.

- Figure 1 would be better positioned appended to the start of Figure 5, and Figures 2 and 3 could be combined (with photos of the device).

- To improve comprehension of the results, summary statistics (ie: means and stdev for main metrics) should be included in the results. For many results only the p-value of the statistical test is presented.

- The authors should describe other open-source wheel designs, such as those created by Bivona et al. 2021 (LOST-Wheel) and Zhu et al. 2021 (WRAQ), the latter of which was published in eNeuro. This should be a dedicated paragraph in the discussion that describes the strengths and limitations of this new wheel in relation to prior open-source wheels. This comment should not be interpreted that the authors need to clarify how their wheel is better than those prior designs, but describe how it compares both positively and negatively.

- Statements regarding simplicity of building and using their system should be tempered. Connecting microcontroller devices to University internet systems is often not simple, and will likely require knowledge of networking and coordination with an IT department. Wording changes in the discussion should more accurately recognize these challenges.

- The statements about battery life of commercial systems lasting <3 weeks is not accurate. Wireless running wheel systems from Actimetrics and Med Associates can last >3 months on battery power.

- There were several minor but necessary corrections noted by R2, which should be addressed in full (I will paste full reviews below).

## Author Response

We appreciate the reviewers and editor’s constructive comments on the manuscript and, in response to this, we have made several changes to the manuscript highlighted in blue font on the manuscript document. We have also addressed each reviewers concern below.

Synthesis of Reviews:

Significance Statement Comments for Author (Required):

The significance statement should be improved to highlight the specific problem being solved by this wheel.

We have added an additional sentence in the significance statement to address the problem being solved by these wheels.

Comments on the Visual Abstract for Author (Required):

N/A

Synthesis Statement for Author (Required):

Essential comments to be addressed in a revision: - The manuscript needs an IACUC (or similar) statement regarding ethical animal use.

We apologize for this oversight - we have added in the ethics statement in the manuscript.

- Photos of the device should be added to Figure 2, ideally within a cage so a reader can easily understand its dimensions.

We have now added photos to figure 2 (now figure 1).

- Figure 1 would be better positioned appended to the start of Figure 5, and Figures 2 and 3 could be combined (with photos of the device).

We have now combined figures 1 and 4 (now figure 2) and have combined figures 2 and 3 (now figure 1).

- To improve comprehension of the results, summary statistics (ie: means and stdev for main metrics) should be included in the results. For many results only the p-value of the statistical test is presented, which does not give an intuitive sense of effect sizes.

We have added means and standard deviations in the results.

- The authors should describe other open-source wheel designs, such as those created by Bivona et al. 2021 (LOST-Wheel) and Zhu et al. 2021 (WRAQ), the latter of which was published in eNeuro. This should be a dedicated paragraph in the discussion that describes the strengths and limitations of this new wheel in relation to prior open-source wheels. This comment should not be interpreted that the authors need to demonstrate how their wheel is better than those prior designs, but rather describe how it compares both positively and negatively.

We have added a paragraph to the discussion discussing the differences between our wheels and other open-source wheels.

- Statements regarding simplicity of building and using their system should be tempered. Connecting microcontroller devices to University internet systems is often not simple, and will likely require knowledge of networking and coordination with an IT department. Minimizing the use of words like "easy" in the discussion would more accurately recognize these challenges.

We have removed statements of simplicity in building the systems in the methods and discussion.

- The statements about battery life of commercial systems lasting <3 weeks is not accurate. Wireless running wheel systems from Actimetrics and Med Associates can last >3 months on battery power, and >1 year in power-saving modes.

We have removed the statement regarding battery power of commercial systems.

- R2 had trouble with dead links, which may have been due to efforts to preserve blinding for review. In the revised manuscript please make sure all links are live.

We have ensured that the links are live and unblinded the github site.

- There were several minor but necessary corrections noted by R2, which should be addressed in full (I will paste full reviews below).

We have addressed these below.

Full reviews:

***********

Reviewer 1

***********

This study shows the development of an open-source open face running wheel for mice that can be used in the home cage. This running wheel records, and stores running data and stores data that can be automatically emailed for further analysis using pipelines provided by the authors. In this study, these running wheels are used to validate an activity-based anorexia model.

Overall, this study is a simple yet robust validation of the usefulness of the running wheels. The open face design of the running wheel makes it compatible with optical experiments, which is an important benefit. Moreover, the documentation of the analysis code and the instructions for assembly is excellent. I find this study suitable for publication to eNeuro. However, I would like to see the following changes in the manuscript:

- The significance statement is weak. A better link between anorexia and mouse models is needed. Currently it reads something like (paraphrasing): "Anorexia is an important health care issue so we developed running wheels for mice.”

We have added the following to the significance statement:

The most used animal model to study AN is the activity-based anorexia model, which measures physical activity during food restriction.

- Please include photos of the actual running wheel in figure 2 that provides some perspective in terms of size and shape.

We have added some photos to figure 2 (now figure 1).

- Lack of battery is a major limitation since it diminishes most of the benefits of being home cage. Although this limitation was addressed, the last sentence dismisses this limitation as unimportant. Please, remove or rephrase the last sentence, since it is a significant limitation to the running wheel, although it still remains very useful despite this limitation.

We have modified the last sentence of the paragraph as follows: “The USB power cable allows for utility despite the battery limitation.”

We have also removed the statement that commercial batteries only last up to 3 weeks.

***********

Reviewer 2

***********

The authors of this manuscript developed an open-source mouse wheel and tested its effectiveness using an activity-based anorexia (ABA) model. The data from testing agrees with both commercial and other open-source designs. This manuscript, however, does not add additional knowledge to the fields of open source hardware nor anorexia-based applications. While previous ABA studies are referenced within the discussion section, they are not directly compared to the data shown here and the reader is left determining themselves if this device and model confirms or refutes existing literature. The authors also fail to describe other open-source designs, such as those created by Bivona et al. 2021 (LOST-Wheel) and Zhu et al. 2021 (WRAQ), the latter of which was published in eNeuro. These two devices have additional features that the authors’ wheel lack.

We added a new paragraph to the discussion comparing the strength and limitations of our design compared to existing open-access wheel running designs in the literature.

This manuscript cannot move forward without the inclusion of an IACUC (or similar) statement. Otherwise, these experiments were conducted without permission and threaten the integrity of all animal research.

We apologize for this mistake. We have added an animal ethics statement that these experiments were covered under our animal care protocol.

Should the authors decide to continue with their submission, major revisions are required to both the manuscript and supporting documentation, and a re-review is requested.

Methods:

Subjects:

•Subjects implies the use of a human model. Please change this heading to "Mice" or "Animals"

We have changed this to mice.

•The the lack of an ethics statement is concerning. This study should have been approved by the appropriate IACUC board. Without approval, this manuscript cannot move forward.

We have added the ethics statement to the methods.

•Latin phrases like ad libitum should be italicized.

We have italicized ad libitum.

ABA model:

•Spinner tops are not mentioned previously and should be clarified if this is the running wheel surface.

We have reordered the results to discuss the construction of the wheels first.

•The authors mention a pilot study, but do not include data. This information would be useful as a supplemental figure.

We have now added this data in the supplement.

Running wheels:

•Raspberry Pi manufacturer information should be included

We have added the supplier for the Raspberry Pi.

•Raspberry Pi is inconsistently capitalized throughout the manuscript

We have capitalized Raspberry Pi throughout the revised manuscript.

3D printing:

•While the inclusion of a markdown file aids the user’s ability to make the wheel, every link on the markdown is a dead link. I suggest the authors migrate their code and files to GitHub. If this was done for the sake of anonymity during the review process and already exists on Github, please disregard this comment.

This file exists on github. We have unblinded the link to allow the reviewer to use the code.

•Software/Printer manufacturers and location are missing.

We have added the manufacturers to the printers and software websites.

Raspberry Pi code:

•The description of the code is logical.

•Does the software exclude accidental movement or movement not by running (rearing onto the wheel)?

The software only records a 1/3rd revolution of the wheel, therefore small accidental movements of the wheel (such a bumping the wheel) are not recorded. We have added this to the methods.

Personal computer:

•Based on the documentation and markdown files on the anonymous site, the authors are skilled programmers. Unfortunately, most individuals are not, and there are many steps that the user will easily make a mistake on. Since MATLAB can be run through Python, I suggest the authors streamline the setup and analyses further, taking advantage of the GUI and packaging the program into a single executable file.

The program for wheel running has been simplified. There are now two GUIs; one that allows you to set up the running wheel system on your computer, similar to an installation, but one needs to run it before doing a new experiment. This set up program is only run once and would not be used for the rest of the experiment. The second GUI is used to run the code to download and analyze the data.

We have provided the Github link in the manuscript (https://github.com/borglandlab/RunningWheel) as the links within appear to become disabled when the repository is anonymized (https://anonymous.4open.science/r/RunningWheel-004D/README.md). We have also listed all the links for the reviewers below.

•Inconsistent capitalization of Python.

We have capitalized Python throughout the revised manuscript.

•I was unable to download the files due to dead links and cannot run the code on my computer.

We have added the github links to the manuscript.

Results:

The statistical evaluation of the wheel running data is extensively covered in the results section, however the actual numbers describing the data are not. The authors should consider including important decreases/increases, weights, distances, velocities, etc. to this section. Figure 1 would be better positioned after Figure 3.

We have combined figure 1 with figure 4 (now figure 2). We have also added in averaged effects for weight, activity during food availability, activity during the postprandial period, as well as velocity during restriction, feeding, and postprandial periods in addition to the averaged data that was already in the manuscript. Figure 4/Page 9:

•The use of "survivability" is misleading. Please revise this figure and accompanying text specify that the curve is the number of mice remaining in the study. If there are data linking the inclusion criteria with survivability, the authors should include it.

We used a Kaplan-Meier survival plot to describe our data. We reworded the results as follows:

"The number of days to reach the humane endpoint whereby mouse body weight dropped to 75% of their body weight from that measured on the last day of baseline was compared between FR exposed to wheel running and FR with a dummy wheel. FR mice exposed to wheel running had reduced probability of survival compared to FR mice with the dummy wheel (Log-rank test: C2 = 5.1, Df = 1, P = 0.02; Figure 2). Thus, FR mice with access to a running wheel are removed from the experiment earlier.” Figure 6/Page 10:

•The mice did not voluntarily drop out from the study. They were removed from the study by the authors. Use of anthropomorphic dialog should be avoided.

We have changed this to mice being removed from the study. Figure 7/Page 12:

•I’m unsure about the conclusion that the mice prefer to run counterclockwise. If the running surface is parallel to the cage bottom, there should not be an effect of direction.

•If the authors would like to substantiate this claim, the wheels should be flipped 180˚ and mice should be retested to see if the counterclockwise behavior is due to a preference or the environment of the cage.

This was only a feature of the wheels that they can measure clockwise or counterclockwise rotation due to the 3 hall sensors. We have contextualized this better that it is a feature of the wheels rather than a mouse behaviour.

"Furthermore, to demonstrate the full utility of the running wheels, total daily activity of both Ad lib and restricted mice was plotted, showing the direction of the rotation of the wheels (i.e., clockwise (CW) or counter clock-wise (CCW); Figure 7B-C). We recorded wheel rotations in CW or CCW from Ad Lib and FR mice, with a greater amount of running in the CCW wheel direction.” Figure 8:

•F and H both contain the same Y axis label. Please revise.

We have fixed this.

Discussion:

The authors discuss the advantages and disadvantages of their wheel and briefly compare it to previous ABA literature. A more extensive review would benefit the reader and add confidence that the device performs as expected.

Additionally, there are other open-source wheels - most recently Bivona et al. 2021, and Zhu et al. 2021 - that are not discussed.

We have added a paragraph discussing the similarities and differences between our wheels and other opensource wheels.

Advantages section:

•Precision of the wheel was not evaluated and the claim that the use of three sensors leads to a high level of precision would need to be determined. Please remove this statement or conduct the appropriate studies.

We have removed this statement.

•After viewing the steps required for data acquisition and analysis, I would disagree that the system is simple, however the hardware aspect to the wheel is of beginner difficulty.

We have removed statements of ease or simplicity.

Disadvantages section:

•I agree that battery power limits many commercial wheels and that the cord is an advantage to the design.

We have removed the statement that commercial batteries last <3 weeks.

•Getting microcontrollers connected to university networks can be a hassle with usernames and passwords constantly changing. Even printers even require IT department approval. Universities like my own specifically bar any device that is not a computer, tablet, or phone. The authors should emphasize that the email portion is a bonus feature and that the data being stored on a microSD is the main collection method.

We have reworded this to: “Second, an added feature of our running wheels is their ability to autonomously collect and transfer data via email in addition to saving the data to an SD card.”

We have also added a statement indicating how we connected the Raspberry Pi’s to the university network.

Below are all the links within our GitHub repository. These links appear to become disabled when the repository is anonymized, but you can access the full repository in the file viewer on the left of the screen. The links are either to a location within the repository or a website (begin with https). running wheel system --> Schematics/Wheel_Schematic.jpg

Base Bottom --> 3Dprintingfiles/STL/Base Bottom.STL

Base Top -->

Sensor Holder -->

Spinner --> 3Dprintingfiles/STL/Base Top.STL

Sensor Holder --> 3Dprintingfiles/STL/Sensor Holder.STL

Spinner --> 3Dprintingfiles/STL/Spinner.STL

Base Top.stl --> 3Dprintingfiles/STL/Base Top.STL

attached images --> 3Dprintingfiles/SupportBlockers/

Base Top.stl --> 3Dprintingfiles/STL/Base Top.STL

attached images --> 3Dprintingfiles/SupportBlockers/

electrical schematic --> Schematics/Electrical_Schematic.jpg

back --> Pictures/RPi_behind.jpg

front --> Pictures/RPi_above.jpg

side --> Pictures/RPi_side.jpg

ApplePiBaker --> https://www.tweaking4all.com/software/macosx-software/applepi-baker-v2/

Raspberry Pi Imager --> https://www.raspberrypi.com/software/

website (MAC address information) --> https://raspberrytips.com/mac-address-on-raspberry pi/

python code for spinner --> Spinner_Code/spinner_WIFI.py/

no wifi spinner code --> Spinner_Code/spinner_noWIFI.py/

completely assembled running wheel --> Pictures/RunningWheel_complete.jpg

macOS --> WheelAnalysis_macOS.md

Windows --> WheelAnalysis_Windows.md

Wheel_Analysis_macOS.md

Anaconda --> https://www.anaconda.com/products/distribution

Miniconda --> https://docs.conda.io/en/latest/miniconda.html

website (about PATH variable) --> https://www.educative.io/answers/how-to-add-python-to-the-path-variable-in-mac

here (about pip) --> https://pip.pypa.io/en/stable/installation/

reference (from MathWorks) -->

https://www.mathworks.com/help/matlab/matlab_external/install-the-matlab-engine-for-python.html

openpyxl --> https://pypi.org/project/openpyxl/

pytz --> https://pypi.org/project/pytz/

matlabengine --> https://www.mathworks.com/help/matlab/matlab_external/install-the-matlab-engine-for-python.html

pyinstaller --> https://pyinstaller.org/en/stable/installation.html

here (for RunningWheel_System_macOS.zip) -->

Analysis_Code/macOS/RunningWheel_System_macOS.zip

here (for RunningWheel_System_ABA_macOS.zip) -->

Analysis_Code/macOS/RunningWheel_System_ABA_macOS.zip

app specific password --> https://support.google.com/mail/answer/185833?hl=en

Wheel_Analysis_Windows.md

Anaconda --> https://www.anaconda.com/products/distribution

website (about PATH variable) --> https://www.educative.io/answers/how-to-add-python-to-path-variable-in-windows

here (about pip) --> https://pip.pypa.io/en/stable/installation/

reference (from MathWorks) -->

https://www.mathworks.com/help/matlab/matlab_external/install-the-matlab-engine-for-python.html

openpyxl --> https://pypi.org/project/openpyxl/

pytz --> https://pypi.org/project/pytz/

matlabengine --> https://www.mathworks.com/help/matlab/matlab_external/install-the-matlab-engine-for-python.html

pyinstaller --> https://pyinstaller.org/en/stable/installation.html

here (for RunningWheel_System_Windows.zip) -->

Analysis_Code/Windows/RunningWheel_System_Windows.zip

here (for RunningWheel_System_ABA_Windows.zip) -->

Analysis_Code/Windows/RunningWheel_System_ABA_Windows.zip

app specific password --> https://support.go
